# S^2^potAE: multimodal spatial spot autoencoder integrating image and transcriptomic features for deconvolution

**DOI:** 10.1093/bib/bbag020

**Published:** 2026-01-31

**Authors:** Tianyi Chen, Wen Xue, Yunfei Zhang, Yongcan Luo, Cheng Liu, Wenjun Shen, Si Wu, Hau-San Wong

**Affiliations:** Department of Computer Science, City University of Hong Kong, Tat Chee Avenue, Kowloon Tong, Kowloon 999077, Hong Kong SAR; School of Computer Science and Engineering, South China University of Technology, 381 Wushan Road, Tianhe District, Guangzhou 510006, Guangdong, P.R. China; School of Future Technology, South China University of Technology, 777 Xingye Avenue East, Panyu District, Guangzhou, 511442, Guangdong, P.R. China; Department of Computer Science, City University of Hong Kong, Tat Chee Avenue, Kowloon Tong, Kowloon 999077, Hong Kong SAR; Department of Computer Science, Shantou University, 243 Daxue Road, Jinping District, Shantou 515063, Guangdong, China; Department of Bioinformatics, Shantou University Medical College, 22 Xinling Road, Jinping District, Shantou 515041, China; School of Computer Science and Engineering, South China University of Technology, 381 Wushan Road, Tianhe District, Guangzhou 510006, Guangdong, P.R. China; Department of Computer Science, City University of Hong Kong, Tat Chee Avenue, Kowloon Tong, Kowloon 999077, Hong Kong SAR

**Keywords:** spatial transcriptomics, autoencoder, graph neural network, multi-scale feature aggregation, histology image analysis, spot deconvolution

## Abstract

Spatial transcriptomics (ST) technologies have significantly advanced our ability to discern gene expression patterns within intact tissue structures, enabling unprecedented insights into cellular heterogeneity and tissue architecture. However, accurately determining cell-type proportions within spatially aggregated transcriptomic spots remains challenging due to inherent granularity discrepancies, batch effects, and spatial heterogeneity. To address these challenges, we introduce S$^{2}$potAE, a novel spatial spot autoencoder framework that integrates gene expression data, spatial coordinates, and morphological features from histology images for precise spot-level deconvolution. S$^{2}$potAE employs a multilevel feature aggregation strategy, systematically extracting and fusing spatially-aware features through a graph-based spatial encoder and perceptual image embeddings from histological patches. Furthermore, an auxiliary pathological classification task enhances biological relevance and model interpretability. Comprehensive benchmarking across multiple simulated and real datasets—including human breast cancer, mouse brain anterior, and human dorsolateral prefrontal cortex—demonstrates that S$^{2}$potAE consistently surpasses state-of-the-art methods in accuracy, robustness, and biological interpretability. Our approach effectively resolves complex cellular compositions, accurately identifies tumor boundaries, and captures nuanced cell-type distributions, significantly enhancing the utility of ST in biological research and clinical applications.

## Introduction

Spatial transcriptomics (ST) represents a groundbreaking advancement in molecular biology, empowering researchers to visualize and quantify gene expression within the native spatial context of intact tissue sections [1–3]. In contrast to conventional transcriptomic approaches—such as bulk RNA sequencing [4–6], which averages signals across diverse cell populations, or single-cell RNA sequencing (scRNA-seq), which dissociates cells and thus loses spatial information—ST uniquely preserves the spatial relationships among cells and their microenvironments [7]. This spatial resolution is critical for inferring the complex molecular architecture of tissues, as it enables the identification of gene expression heterogeneity not only at the cellular level but also in relation to tissue structure and organization.

Despite rapid technological advances, current ST platforms [2, 8] such as 10x Genomics Visium [9], Slide-seq [10], and MERFISH [11] differ in spatial resolution and throughput, with most spot-based methods capturing gene expression from multiple neighboring cells per spot [12, 13]. While this limits single-cell resolution, it preserves tissue architecture, enabling the study of spatially organized biological processes. In complex environments like the tumor microenvironment, ST facilitates the mapping of cell–cell interactions and the identification of spatial gene expression patterns underlying disease [14, 15]. ST technologies have become essential tools for uncovering new insights into tissue organization and pathology.

However, accurately deciphering the cellular composition within ST data remains a substantial computational and analytical challenge. Each spatially resolved “spot” in ST data typically aggregates gene expression profiles from multiple heterogeneous cells, obscuring precise cell-type proportions within these spots [16, 17], thus reliably decomposing spot-level gene expression data into constituent cell-type proportions—known as the cell-type deconvolution task. This is essential for accurate downstream biological interpretation [18, 19]. However, several inherent challenges complicate this task. First, the aggregated nature of ST spots creates significant granularity discrepancies with scRNA-seq data, commonly used as reference datasets for deconvolution [20, 21]. Second, batch effects, technical variations, and biological heterogeneity between ST and scRNA-seq modalities introduce substantial discrepancies, further complicating accurate and robust inference of cell-type composition [22, 23].

In recent years, numerous computational methods have been developed to address the deconvolution of ST data. Typically, these methods leverage scRNA-seq data as reference profiles to infer cellular compositions within ST spots [22, 24, 25]. For instance, Tangram employs deep integration of scRNA-seq and ST data, creating a probabilistic mapping to infer cell-type proportions [17]. GraphST utilizes graph-based representations to capture spatial dependencies among neighboring spots, facilitating improved inference of cell compositions [26]. Cell2Location employs Bayesian hierarchical models to probabilistically assign cell-type compositions to ST spots, thereby improving robustness in complex tissues [16]. Additionally, generative adversarial network-based methods propose domain alignment between data modalities to mitigate batch effects and enhance accuracy [23]. Spadecon [22] demonstrates superior performance across multiple benchmark datasets, providing enhanced computational efficiency and accuracy compared with prior methods. Similarly, MACD [24] proposes a masked adversarial neural network framework to effectively align real ST data with simulated ST data derived from scRNA-seq references. Despite these advancements, most existing approaches still inadequately address the inherent spatial heterogeneity and structural complexity within tissues. These limitations typically result in suboptimal accuracy when analyzing tissues characterized by strong spatial dependencies or structured cellular organization, such as tumor samples or complex tissue architectures.

To effectively address the aforementioned limitations, we propose an innovative multimodal **S**patial **Spot A**uto**E**ncoder structure, named S$^{2}$potAE, designed specifically to enhance ST spot deconvolution accuracy. Our proposed method explicitly incorporates spatially informative priors derived from both spatial coordinates and tissue morphology, thus significantly improving the interpretability and accuracy of cellular composition inference. Specifically, S$^{2}$potAE leverages spatial coordinate information to condition the input gene expression features, enabling location-aware modeling of ST spot data. Furthermore, we integrate large-scale pathological histology images associated with ST data, extracting perceptual image features at the spot-level to provide critical morphological context. These enriched visual and spatial features are systematically integrated via a carefully designed attention-based decoder architecture. The attention mechanism selectively guides the model to reconstruct and predict cellular proportions within each spot, significantly enhancing predictive performance by incorporating tissue morphology and spatial context. Moreover, we introduce an auxiliary prediction task aimed at distinguishing diseased from non-diseased tissue regions within each spot. This auxiliary supervision provides additional biologically relevant constraints, enabling better representation learning and more accurate decomposition of cellular compositions, especially within complex heterogeneous tissues. By comprehensively integrating biological knowledge, spatial priors, and morphological context into an end-to-end deep learning framework, S$^{2}$potAE substantially advances the accuracy and interpretability of ST deconvolution. Ultimately, our method facilitates deeper biological insights into the structural and functional organization of tissues, significantly enhancing the utility and interpretability of ST data in complex biological and clinical research scenarios.

## Methods and materials

### Methods

To formalize the data flow and tasks in our study, we first define the key notations and inputs. The process of generating pseudo-spot-based simulated ST data from scRNA-seq data is adapted from the approach described in [21]. Specifically, pseudo-spots are generated by aggregating transcriptomic profiles from single cells obtained in scRNA-seq datasets. This simulation process enables the construction of pseudo-spot-based simulated ST data, which serves as a valuable resource for benchmarking and model training in ST studies.

Let the simulated ST data matrix be denoted as ${X}_{\mathrm{simu}} \in \mathbb{R}^{S_{1} \times G}$, where $S_{1}$ represents the number of simulated spatial spots (a parameter that can be specified during simulation), and $G$ denotes the number of genes profiled. Similarly, the real ST data matrix is denoted as ${X}_{\mathrm{real}} \in \mathbb{R}^{S_{2} \times G}$, where $S_{2}$ represents the number of spatial spots in the real dataset. For real ST data, spatial coordinates for each spot are provided in the form of a matrix ${Y}_{\mathrm{real}} \in \mathbb{R}^{S_{2} \times 2}$, where the second dimension encodes the Cartesian coordinates relative to the spatial grid. These coordinates are typically derived from Hematoxylin and Eosin (H&E) stained histological images.

In addition to the ST data, simulated ST data are associated with cell-type proportion labels, represented by a matrix ${Y}_{\mathrm{prop}} \in \mathbb{R}^{S_{1} \times P_{1}}$. Here, $P_{1}$ denotes the number of distinct cell types modeled in the simulation. For real ST data, spot-level pathological annotations are provided in a matrix ${Y}_{\mathrm{spot}} \in \mathbb{R}^{S_{2} \times P_{2}}$, where $P_{2}$ corresponds to the number of pathological categories or classes.

#### Backbone overview

**Figure 1:**
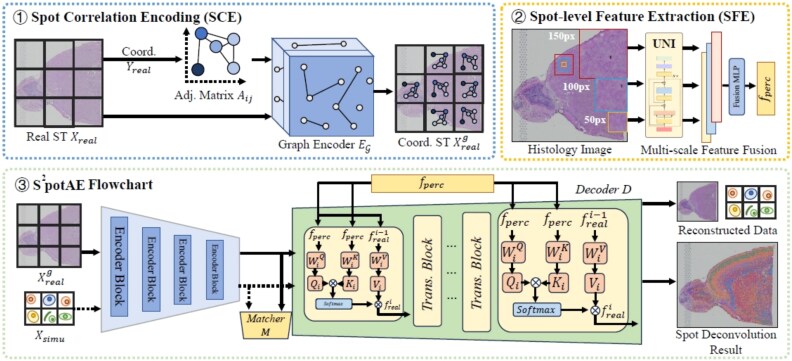
Overview of the S²potAE framework and its data flow.

The main components of S$^{2}$potAE consist of five key modules: the encoder $E$, the multimodal conditional decoder $D$, the graph encoder $E_{\mathcal{G}}$, multitask predictors (including the spot proportion predictor $P_{\mathrm{prop}}$ and the spot type predictor $P_{\mathrm{spot}}$), and the domain matcher $M$. Each module is designed to work cohesively, addressing the complex requirements of ST data analysis. The integration of these components enables S$^{2}$potAE to simultaneously address data reconstruction, cell-type proportion prediction, and pathological classification tasks. The detailed data flow and interactions among these modules are illustrated in Fig. 1. This modular design not only ensures flexibility but also facilitates the seamless integration of diverse data modalities, leading to robust and interpretable predictions in ST analysis.

#### Modeling spatial dependencies via graph encoding

To capture the spatial relationships between spots in the real ST data, we leverage the positional information encoded in ${Y}_{\mathrm{real}}$. The expression levels of spatially adjacent spots exhibit positional correlations, which we model using a graph-based representation. Specifically, we construct an adjacency matrix ${A}_{ij}$ by identifying the $k$-nearest neighbors (KNN) for each spot. For each spot $i$, edges are created to connect it with its $k = 5$ nearest neighbors. The adjacency matrix ${A}_{ij}$ is defined as


(1) \begin{align*}& A_{ij} = \begin{cases} 1, & \mathrm{if}\ j \in \mathrm{KNN}(i), \\ 0, & \mathrm{otherwise}. \end{cases}\end{align*}

To ensure numerical stability and mitigate the risk of oversmoothing during graph convolution, we normalize the adjacency matrix using a degree matrix $\hat{D}$, where $\hat{D}_{ii} = \sum _{j} \hat{A}_{ij}$, and $\hat{A}_{ij} = A_{ij} + I$. The addition of the identity matrix $I$ introduces self-loops, allowing each node to retain its original features during graph convolution. At this point, we define this process as the “Spot Correlation Encoding (SCE)” module. This module constructs a graph representation of the ST data and encodes it using a graph encoder $E_{\mathcal{G}}$. The graph encoder propagates and transforms node features through multiple graph convolutional layers. The forward propagation for the $(l+1)$ th layer is expressed as:


(2) \begin{align*}& H^{(l+1)} = \sigma\left(\hat{D}^{-\frac{1}{2}} \hat{A} \hat{D}^{-\frac{1}{2}} H^{(l)} W^{(l)}\right),\end{align*}

where $H^{(l)} \in \mathbb{R}^{R \times d^{(l)}}$ represents the node feature matrix at layer $l$ (with $H^{(0)} = X_{\mathrm{real}}$ as the initial input features), $W^{(l)} \in \mathbb{R}^{d^{(l)} \times d^{(l+1)}}$ is the learnable weight matrix for the $l$th graph convolution layer, and $\sigma (\cdot )$ denotes the ReLU activation function. The output of the final layer, $X_{\mathrm{real}}^{g}$, encodes both gene expression profiles and spatial positional information. The graph-encoded features, $X_{\mathrm{real}}^{g}$, are subsequently passed into the encoder $E$ to generate intermediate representations, denoted as $f_{\mathrm{real}}^{g}$. These representations are then fed into the decoder $D$ for reconstructing the ST matrix. During the decoding phase, positional encoding structures are incorporated into the conditional decoder to ensure that the reconstructed ST matrix retains spatial coherence. To accommodate the variable gene dimensions across datasets, we adopt the maximum gene length observed in each dataset as the embedding dimension for the positional encoder. By integrating graph-based learning and coordinate-based embeddings, the SCE module effectively learns spatially aware features, significantly enhancing the performance of the spot deconvolution task.

#### Spot-level feature extraction and fusion

ST data are accompanied by paired H&E-stained histological images, which provide vital morphological context for interpreting spatial gene expression. To harness this, we introduce a Spot-level Feature Extraction (SFE) module (see Fig. 1). For each spot in the ground-truth data, $Y_{real}$, we extract multi-scale image patches $\mathcal{P}_{s}$ centered at the spot, with $s$ denoting different patch sizes (e.g. $\mathcal{P}_{150}$, $\mathcal{P}_{100}$, $\mathcal{P}_{50}$). For each patch $\mathcal{P}_{s}$, we obtain its token-level features by feeding it into the **UNI** [27] encoder:


(3) \begin{align*}& f_{UNI}^{(i)} = \mathrm{UNI}(\mathcal{P}_{s}^{(i)}),\end{align*}

where $f_{UNI}^{(i)} \in \mathbb{R}^{N_{s} \times d}$ denotes the $N_{s}$ token embeddings of dimension $d$ for the $i$th scale. Collecting outputs from all scales, we get


(4) \begin{align*}& \mathbb{U}_{f_{uni}} = \mathrm{UNI}(\mathcal{P}_{s}),\end{align*}

where $\mathbb{U}_{f_{uni}} \in \mathbb{R}^{3 \times N_{s} \times d}$, with $N_{s}$ tokens of dimension $d$ for each scale. To obtain compact representations, we apply self-attention pooling [28] at each scale:


(5) \begin{align*}& p_{s} = \mathrm{AttnPool}(\mathbb{U}_{f_{uni}}) = \mathrm{softmax}\left(\frac{W_{(i)} f_{UNI}^{(i)}}{\sqrt{d}}\right) f_{UNI}^{(i)},\end{align*}

where $W_{(i)} \in \mathbb{R}^{d}$ are learnable pooling weights. This process yields concise, informative embeddings that integrate multi-scale morphological context into downstream transcriptomic analyses. Subsequently, we design a specialized multilayer perceptron (MLP)-based fusion structure to integrate these multiscale representations into a unified latent spot-level perceptual feature embedding. The fusion process can be formalized as follows:


(6) \begin{align*}& f_{perc} = F_{fuse}\left(\mathrm{concat}(p_{s})\right),\end{align*}

where $F_{fuse}(*)$ is a three-layer MLP equipped with ReLU activation functions, effectively capturing complex inter-scale correlations and generating a unified perceptual representation $f_{perc} \in \mathbb{R}^{d}$. To inject these perceptual priors into the conditional decoding phase of our model, we further modify the decoder $D$ to incorporate an attention-based fusion strategy. Specifically, perceptual embeddings serve as the Key and Value vectors in a transformer-based multi-head attention block, while intermediate-level features from the ST data ${f}^{g}_{real}$ serve as Query. This cross-modal attention mechanism is mathematically represented as


(7) \begin{align*}& \begin{aligned} \mathrm{Attn}(Q, K, V) = \mathrm{softmax}\left(\frac{QK^{T}}{\sqrt{d_{k}}}\right)V, \\ Q = f^{g}_{real} W_{Q}, K = f_{perc}W_{K}, V = f_{perc}W_{V}, \end{aligned}\end{align*}

where $W_{Q}, W_{K}$, and $W_{V}$ are, respectively, the learnable linear transformation matrices projecting the Query, Key, and Value vectors into the common attention space with dimension $d_{k}$. The resulting spot-level perceptual features $f_{perc}$, obtained through the proposed SFE module, are subsequently integrated into every decoding layer of the conditional decoder $D$. This integration serves as a cross-modal conditioning strategy, enabling the decoder to dynamically leverage histological visual context at multiple decoding stages. Through this design, our model effectively captures and exploits intricate morphological patterns and spatially resolved tissue characteristics, enhancing the interpretability and predictive accuracy of the ST deconvolution task.

#### Spot pathological classification as an auxiliary task for deconvolution

The fundamental goal of the spot deconvolution task is to infer the proportional cellular composition of individual spatial spots, quantifying the specific mixture of cell types or pathological components within each spatial location. For simulated ST data, the simulated proportional composition of pathological regions within each spot is represented by ${Y}_{\mathrm{prop}} \in \mathbb{R}^{S_{1} \times P_{1}}$. Formally, given simulated ST data $X_{sim}$, the Encoder $E$ in S$^{2}$potAE generates intermediate latent representations as follows:


(8) \begin{align*}& f_{latent}^{simu} = E(X_{simu}), \quad f_{latent} \in \mathbb{R}^{S_{1} \times d_{f}},\end{align*}

where $d_{f}$ denotes the dimensionality of the latent embedding space. Meanwhile, we propose an auxiliary task named Spot Pathological Classification (SPC), explicitly leveraging spot-level pathological annotations to complement the primary deconvolution task. Specifically, we utilize annotated pathological labels from real ST images, denoted as ${Y}_{hist} \in \mathbb{R}^{S_{2} \times P_{2}}$. Incorporating explicit spot-level annotations enables the model to implicitly encode histological distinctions and pathological characteristics, thereby enhancing both interpretability and predictive accuracy. The latent representations of annotated spots from real ST data are similarly obtained via the encoder $E$:


(9) \begin{align*}& f_{latent}^{hist} = E(X_{real}), \quad f_{latent}^{hist} \in \mathbb{R}^{S_{2} \times d_{f}}.\end{align*}

Recognizing the complementary nature of these two tasks, we introduce a carefully designed three-layer MLP as a shared feature extractor $F_{shared}$:


(10) \begin{align*}& \begin{aligned} f_{shared}^{simu} = F_{shared} \left(f_{latent}^{simu}\right), \ f_{shared}^{hist} = F_{shared} \left(f_{latent}^{hist}\right), \end{aligned}\end{align*}

where $d_{s}$ denotes the dimensionality of the shared latent feature space. These shared embeddings are subsequently utilized by their respective prediction heads to enforce implicit alignment and cross-task feature consistency:

(1) For the spot deconvolution task, the pathological proportion predictor $C_{prop}$ outputs the estimated proportional composition of each spot:


(11) \begin{align*}& \hat{Y}_{prop} = C_{prop} \left(f_{shared}^{simu}\right).\end{align*}

The primary deconvolution loss $\mathcal{L}_{deconv}$ is defined as a categorical cross-entropy loss:


(12) \begin{align*}& \mathcal{L}_{deconv} = -\sum_{i=1}^{S_{1}}\sum_{j=1}^{P_{1}} Y_{prop, ij}\log(\hat{Y}_{prop, ij}).\end{align*}

(2) For the auxiliary spot pathological classification task, the pathological classification head $C_{hist}$ predicts the pathological class for each annotated spot:


(13) \begin{align*}& \hat{Y}_{hist} = C_{hist}\left(f_{shared}^{hist}\right).\end{align*}

Correspondingly, the auxiliary classification loss $\mathcal{L}_{hist}$ is also formulated using categorical cross-entropy:


(14) \begin{align*}& \mathcal{L}_{hist} = -\sum_{i=1}^{S_{2}}\sum_{j=1}^{P_{2}} Y_{hist, ij}\log(\hat{Y}_{hist, ij}).\end{align*}

In summary, integrating the auxiliary SPC task with spot deconvolution creates a synergistic framework that leverages shared latent features. This approach improves both the accuracy of cell-type proportion estimates and the interpretability of pathological classifications, leading to more robust and biologically meaningful predictions.

#### Overall losses and optimization

To comprehensively optimize the proposed S$^{2}$potAE framework, we integrate multiple complementary loss functions that collectively enhance the performance, interpretability, and generalization capacity of the model across distinct ST data domains. First, to ensure accurate reconstruction of spatial gene expression patterns, we adopt a mean squared error (MSE)-based reconstruction loss. This loss quantitatively assesses the discrepancy between the reconstructed expression data generated by the conditional decoder $D$ and the corresponding real ST data, promoting faithful recovery of the underlying biological signals:


(15) \begin{align*}& \mathcal{L}_{recon} = \sum_{i=1}^{R} \sum_{j=1}^{G} [X_{pred, ij} - D(f^{g}_{real} | f_{perc}) ]^{2},\end{align*}

where $X_{pred}$ is the reconstructed ST data, $f^{g}{real}$ are latent features from real ST data, $f{perc}$ is the perceptual embedding from histological image patches, and $R$ and $G$, respectively, denote the numbers of spatial spots and genes. To address distributional differences between simulated and real ST data, we employ a gradient reversal layer (GRL)-based domain discriminator $M$, promoting domain-invariant feature learning through adversarial training. This enhances the cross-domain generalizability and biological consistency of the latent representations. The domain discrimination loss is defined as a binary cross-entropy objective:


(16) \begin{align*}& \begin{aligned} \mathcal{L}_{match} = -\frac{1}{R} \sum_{i=1}^{R} [ c_{i} &\log(M(f^{g}_{real})) \\ + (1-c_{i}) &\log(1 - M(f_{simu})) ], \end{aligned}\end{align*}

where $f_{simu}$ indicates the simulated ST data embeddings extracted by encoder $E$ and passed through the GRL, $c_{i}$ is a binary indicator of the data domain (real or simulated), and $R$ denotes the aggregate number of training samples across domains. The total training loss for the S$^{2}$potAE architecture integrates all defined loss components into a unified optimization target:


(17) \begin{align*}& \mathcal{L}_{total} = \mathcal{L}_{deconv} + \lambda_{1} \cdot \mathcal{L}_{hist} + \lambda_{2} \cdot \mathcal{L}_{recon} + \lambda_{3} \cdot \mathcal{L}_{match},\end{align*}

where $\mathcal{L}_{total}$ denotes the total task loss. Hyperparameters $\lambda _{1}$, $\lambda _{2}$, and $\lambda _{3}$ balance the relative importance of each loss term to ensure stable and effective training. All constituent sub-networks within the model are jointly trained from scratch through end-to-end optimization.

### Experimental settings and benchmarks

We evaluate our method by comparing it with eight state-of-the-art spot deconvolution approaches developed in recent years, including Tangram [17], GraphST [26], Spoint [21], Cell2location [16], DestVI [20], scpDeconv [23], SpaDeconv [22], and MACD [24]. Following Tangram, we adopt a $K$-fold ($K=10$) cross-validation strategy to train and evaluate our model. Specifically, for each dataset, we randomly partition the spatial spots into 10 equally sized folds. During each iteration, one fold is held out as the test set, while the remaining nine folds are used for training. This process is repeated 10 times, such that each fold serves as the test set exactly once. Model performance is then averaged across all 10 folds to provide a robust estimate of generalization.

**Implementation details:** All baseline models are obtained from their official GitHub repositories, ensuring reproducibility and consistency. The computational experiments were conducted on an RTX 4090 GPU using PyTorch version 1.10. Notably, our model demonstrates robustness across different datasets without requiring extensive tuning. The hyperparameters used for training across different datasets, including learning rates, number of epochs, batch size, and specific parameter values, are summarized in Table 1. For each dataset, we began with widely used default values with previous work [24]. For the auxiliary loss coefficients ($\lambda _{1}, \lambda _{2}, \lambda _{3}$), we adopted values inspired by previous works [29], with additional fine-tuning based on validation performance. In most cases, we observed that the model was relatively insensitive to small changes in these parameters, and thus the selected values serve as robust defaults. We adopted values based on established practices in the literature, making minor adjustments only if necessary to improve validation performance. Overall, we found that the model’s performance was generally robust to small changes in these parameters, and the values in Table 1 can be regarded as effective defaults for a wide range of scenarios.

**Table 1:** Overview of hyperparameters utilized across different datasets, including learning rates, number of epochs, and specific parameter values.

Hyperparameters	Benchmarks			
	Simulated	10x_HBC	10x_MBA	DLPFC (151673)
Learning rate	1e-2	1e-3	1e-3	1e-3
Epochs	500	300	400	300
Batch size	256	400	400	400
Hyperparameter $\lambda _{1}$	0.0	0.1	0.1	0.1
Hyperparameter $\lambda _{2}$	0.1	0.1	0.1	0.1
Hyperparameter $\lambda _{3}$	0.0	0.01	0.01	0.01

To process histology images, we cropped and resized patches of size $224 \times 224$ pixels centered around each spatial spot to ensure uniform input dimensions for the model. This patch size was selected to balance computational efficiency and the preservation of local pathological information. To evaluate model performance, we used three quantitative metrics: Pearson correlation coefficient (PCC), structural similarity index measure (SSIM), and root mean squared error (RMSE). For each spot in the test set, we compared the predicted gene expression profile with the ground truth.

- PCC was calculated between the predicted and true gene expression vectors for each spot, then averaged across all spots in the test set. For each spot (or gene), PCC is computed as


\begin{align*} & \begin{array}{l} \mathrm{PCC}(X_{\mathrm{real}}, X_{\mathrm{pred}}) \\[1.5ex] = \frac{ \sum_{i=1}^{n} (X_{\mathrm{real}}^{i} - \bar{X}_{\mathrm{real}}) (X_{\mathrm{pred}}^{i} - \bar{X}_{\mathrm{pred}}) }{ \sqrt{\sum_{i=1}^{n} (X_{\mathrm{real}}^{i} - \bar{X}_{\mathrm{real}})^{2}} \sqrt{\sum_{i=1}^{n} (X_{\mathrm{pred}}^{i} - \bar{X}_{\mathrm{pred}})^{2}}, } \end{array} \end{align*}

where ${X}_{\mathrm{pred}}$ denotes the predicted expression values, and $\bar{X}_{\mathrm{real}}$, $\bar{X}_{\mathrm{pred}}$ are their respective means.

- SSIM was used to assess the similarity between the predicted and true spatial expression patterns for each gene, and the average SSIM across all genes was reported.

Given two spatial expression matrices ${X}_{\mathrm{real}}$ and ${X}_{\mathrm{pred}}$, SSIM is calculated as


\begin{align*} & \begin{array}{l} \mathrm{SSIM}(X_{\mathrm{real}}, X_{\mathrm{pred}}) \\[1.5ex] = \frac{ (2\mu_{X_{\mathrm{real}}} \mu_{X_{\mathrm{pred}}} + C_{1}) (2\sigma_{X_{\mathrm{real}} X_{\mathrm{pred}}} + C_{2}) }{ (\mu_{X_{\mathrm{real}}}^{2} + \mu_{X_{\mathrm{pred}}}^{2} + C_{1}) (\sigma_{X_{\mathrm{real}}}^{2} + \sigma_{X_{\mathrm{pred}}}^{2} + C_{2}), } \end{array} \end{align*}

where $\mu _{X_{\mathrm{real}}}$, $\mu _{{X_{\mathrm{pred}}}}$ are the means, $\sigma _{X_{\mathrm{real}}}^{2}$, $\sigma _{{X_{\mathrm{pred}}}}^{2}$ are the variances, $\sigma _{{X_{\mathrm{real}}}{X_{\mathrm{pred}}}}$ is the covariance of $\mathbf{{X_{\mathrm{real}}}}$ and $\mathbf{{X_{\mathrm{pred}}}}$, and $C_{1}, C_{2}$ are small constants to stabilize the division.

- RMSE was computed between the predicted and true gene expression profiles for each spot and then averaged over all test spots. The RMSE between the predicted and ground truth values is computed as


\begin{align*} & \mathrm{RMSE}({X_{\mathrm{real}}}, {X_{\mathrm{pred}}}) = \sqrt{ \frac{1}{n} \sum_{i=1}^{n} ({X_{\mathrm{pred}}}^{i} - {X_{\mathrm{real}}}^{i})^{2}}, \end{align*}

where $n$ is the number of elements (e.g. genes or spots). After completing the 10-fold cross-validation, we concatenated the predictions from all test folds and computed the final PCC, SSIM, and RMSE values on the aggregated results. This ensures that each sample is evaluated exactly once in the held-out setting, and the reported metrics reflect the overall model performance on the entire dataset.

**Benchmarks:** We conducted extensive evaluations on 32 simulated datasets [30], where single-cell data were derived from real tissue samples, and the corresponding ST data were generated following the approach described in [21]. Specifically, to simulate pseudo-spots, cells were sampled from an scRNA-seq dataset according to predefined probabilistic rules that reflect the cell type composition at each spot. The number of cells per spot ($N_{c}$) was drawn from a normal distribution, and the number of cell types per spot ($N_{t}$) was also drawn from a normal distribution with parameters set as in [21]. For each spot, cells of each type were randomly sampled based on their proportions in the scRNA-seq data, and the gene expression profiles of these sampled cells were aggregated to generate the pseudo-spot expression. The cell type labels for each spot were recorded according to the sampled composition. Finally, to further mimic the characteristics of real ST data, the gene expression matrix was downsampled using the downsampleMatrix function from the Scuttle package, resulting in simulated spots with gene expression distributions similar to those observed in actual ST data. In addition, we tested our method on three real pathological slide datasets, which offer diverse and challenging benchmarks for validation:

1. 10x_HBC (10X Visium Human Breast Cancer) [31]: this dataset contains 3798 ST spots with 36 601 genes, providing detailed molecular insights into human breast cancer pathology.

2. 10x_MBA (10X Visium Mouse Brain Anterior) [26]: this dataset includes 2695 ST spots and 32 285 genes, capturing the transcriptomic landscape of the mouse brain anterior region.

3. LIBD DLPFC (Human Dorsolateral Prefrontal Cortex) [32]: this dataset consists of spatially resolved transcriptomic profiles from 12 slices of the dorsolateral prefrontal cortex (DLPFC). We specifically used slice 151673, which contains 3639 ST spots and 33 538 genes. The dataset provides a comprehensive view of cortical layers and white matter.

4. Slide-seqV2_Hippocampus (Mouse Hippocampus) [26]: this dataset was acquired using the Slide-seqV2 platform and consists of 52 869 spatial spots and 23 264 genes from mouse hippocampus section Puck_200115_08, providing near single-cell resolution for comprehensive spatial transcriptomic analysis.

5. MERFISH_Hypothalamus (Mouse Hypothalamic Preoptic Area) [11, 33]: this dataset comprises 5557 spatial spots and 155 genes from a mouse hypothalamic preoptic area slice (Bregma –0.04 mm) generated by the MERFISH platform, enabling high-resolution profiling of fine-scale brain structures.

These benchmarks are selected to evaluate our method on datasets with varying tissue types, spatial resolutions, and biological complexities, ensuring a thorough assessment of its generalizability and performance.

## Results

### Robust spot deconvolution using S$^{2}$potAE

In this section, we evaluate the performance of S$^{2}$potAE in spot-level deconvolution tasks across both simulated and real ST datasets. As shown in Table 2, our method consistently outperforms other state-of-the-art techniques, demonstrating superior reconstruction accuracy and biological fidelity. Across the 32 simulated datasets, S$^{2}$potAE achieved a mean PCC of 0.7951, surpassing the second-best method, MACD, by a margin of 0.0197. Notably, S$^{2}$potAE not only excelled in correlation metrics but also exhibited the lowest RMSE and highest SSIM, highlighting its ability to effectively preserve both global and local spatial features. For real datasets, including 10x_HBC, 10x_MBA, and DLPFC (slice 151673), our method demonstrated robust generalizability. The PCC values for S$^{2}$potAE were consistently higher than those of other methods, with improvements of up to 0.0340 compared with the second-best approach. This indicates that S$^{2}$potAE can reliably infer cell-type proportions and reconstruct ST profiles, even in complex real-world datasets. While MACD performed well on simulated datasets, its performance was less reliable on real datasets, likely due to domain-specific discrepancies. GraphST and Spoint, although stable across datasets, showed lower PCC and SSIM values, suggesting limitations in capturing fine-grained spatial heterogeneity. In contrast, S$^{2}$potAE effectively bridged this gap by integrating simulated and real datasets into a unified framework, ensuring both accuracy and consistency.

**Table 2:** Quantitative results with competing methods on Simulated Data, 10x_HBC, 10x_MBA, and DLPFC (slice 151673). The table compares three key metrics: PCC$\uparrow $, SSIM$\uparrow $, and RMSE$\downarrow $. For PCC and SSIM, higher values indicate better performance ($\uparrow $), as they reflect stronger correlation or similarity with the ground truth. Conversely, for RMSE, lower values ($\downarrow $) are preferred, as they indicate smaller reconstruction errors. Best results for each dataset are highlighted in **bold**, while second-best results are underlined. These metrics collectively evaluate the accuracy, structural fidelity, and error minimization of the competing methods across diverse ST datasets.

Simulated data			
Methods	PCC$\uparrow $	SSIM$\uparrow $	RMSE$\downarrow $
Tangram [17]	0.7315	0.6884	0.1441
GraphST [26]	0.7441	0.7210	0.1021
Spoint [21]	0.7101	0.6754	0.1211
Cell2location [16]	0.6889	0.4889	0.1448
DestVI [20]	0.4412	0.4984	0.1458
scpDeconv [23]	0.5984	0.4574	0.1321
SpaDeconv [22]	0.6415	0.5512	0.1120
MACD [24]	0.7754	0.7110	0.0984
Ours	**0.7951**	**0.7265**	**0.0884**
**10x_HBC**			
Tangram [17]	0.1829	0.0530	1.2736
GraphST [26]	0.2101	0.1542	1.2432
Spoint [21]	0.1663	0.0044	1.2868
Cell2location [16]	-	-	-
DestVI [20]	0.1837	0.0532	1.2730
scpDeconv [23]	0.1446	0.0641	1.2732
SpaDeconv [22]	-	-	-
MACD [24]	0.2015	0.0884	1.2458
Ours	**0.2441**	**0.2541**	**1.1032**
**10x_MBA**			
Tangram [17]	0.1847	0.0994	1.2841
GraphST [26]	0.2223	0.1788	1.2211
Spoint [21]	0.1896	0.1021	1.2654
Cell2location [16]	-	-	-
DestVI [20]	0.1845	0.1121	1.2675
scpDeconv [23]	0.1654	0.0645	1.3546
SpaDeconv [22]	-	-	-
MACD [24]	0.1754	0.0655	1.2987
Ours	**0.2498**	**0.2015**	**1.1215**
**DLPFC (slice 151673)**			
Tangram [17]	0.1754	0.1544	1.3994
GraphST [26]	0.2210	0.1754	1.3315
Spoint [21]	0.1321	0.1021	1.4786
Cell2location [16]	-	-	-
DestVI [20]	0.0823	0.0043	1.4541
scpDeconv [23]	0.1554	0.1421	1.4451
SpaDeconv [22]	-	-	-
MACD [24]	0.2254	0.1874	1.3542
Ours	**0.2333**	**0.1987**	**1.3215**

### Spatially resolved spot deconvolution in real-world datasets using S$^{2}$potAE

**Figure 2:**
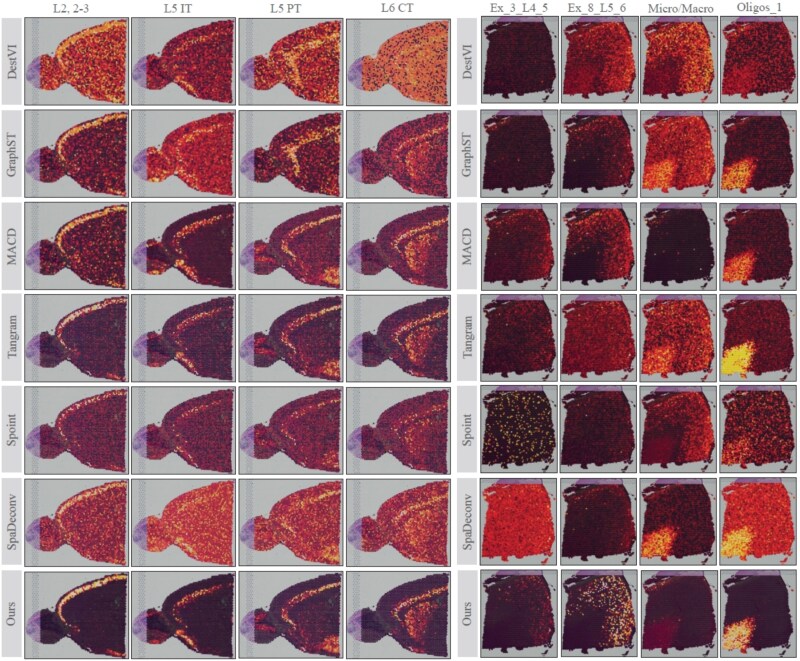
Comparative evaluation of deconvolution methods across cortical layers and cell types. The figure illustrates a comprehensive comparison of the performance achieved by deconvolution methods across distinct cortical neuronal layers (“L2, 2-3”, “L5 IT”, “L5 IT,” and “L6 CT”) and an overall assessment within the 10x_MBA dataset (first four columns). Additional assessments are provided for selected cellular composition categories (“Ex_3_L4_5,” “Ex_8_L5_6,” “Micro/Macro,” and “Oligos_1”) within the DLPFC dataset (last four columns).

In this section, we thoroughly evaluate the performance of S$^{2}$potAE in spatially resolved spot deconvolution using two real-world ST datasets: the mouse brain atlas dataset (10x_MBA) and the human dorsolateral prefrontal cortex dataset (DLPFC, slice 151673). As illustrated in Fig. 2, our proposed method demonstrates enhanced accuracy and biological interpretability compared with existing deconvolution methods, particularly in the precise classification of cortical layers and accurate identification of white matter regions.

**Cortical layer deconvolution in the 10x_MBA dataset.**

The 10x_MBA dataset features clearly defined neuronal layers, corresponding to distinct cortical regions within the mouse brain. The inherent spatial organization of these layers provides a biologically meaningful context for assessing computational deconvolution methods. S$^{2}$potAE accurately reconstructs the spatial distribution of these neuronal layers, aligning closely with the corresponding anatomical structures observed in histological H&E-stained images. Notably, for each layer, our method precisely aggregates neuronal populations within their expected cortical regions, significantly reducing misclassification errors. In contrast, competing approaches, such as GraphST, provide reasonable predictions but frequently generate false-positive signals in unrelated areas, thereby compromising their biological relevance. Similarly, MACD successfully identifies neuronal layers but lacks clarity in spatial boundaries, highlighting limitations in resolving finer cortical structures.

**White matter and cellular composition analysis in the DLPFC dataset.** The DLPFC dataset offers a unique and challenging scenario to evaluate deconvolution performance within complex human brain tissues, particularly in white matter regions and adjacent cortical layers. Our method distinctly outperforms existing approaches, including Tangram, GraphST, Spoint, and SpaDeconv, in accurately predicting cellular compositions and delineating their spatial distributions. For example, within the “Ex_3_L4_5” category, competing methods tend to produce dispersed and ambiguous spatial patterns, lacking precision in localizing biologically relevant spots. Tangram and Spoint exhibit diffuse predictions, while SpaDeconv frequently misassigns cellular compositions to unrelated cortical regions. Moreover, within challenging regions such as white matter, our method demonstrates superior accuracy in deconvoluting critical cell types, including “Micro/Macro” and “Oligos_1.” The heatmaps clearly illustrate the enhanced spatial resolution provided by S$^{2}$potAE, highlighting its exceptional capacity to accurately identify and localize cell populations cellular differentiationg consistency across adjacent tissue regions. This robust performance underscores S$^{2}$potAE’s potential in effectively resolving cellular heterogeneity within complex human brain structures.

**Figure 3:**
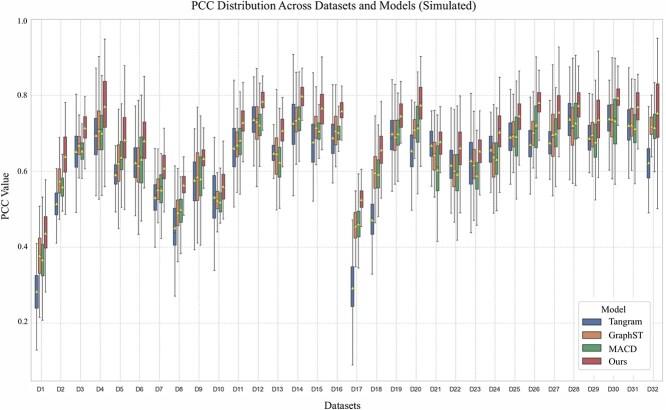
Comparison of PCC (Pearson Correlation Coefficient) distributions across different datasets and models in a simulated scenario. The models compared include Tangram, GraphST, MACD, and Ours. The results demonstrate the robustness of our approach across a wide range of datasets.

In addition, Fig. 3 quantitatively evaluates the robustness and comparative accuracy of S$^{2}$potAE through PCC distributions across 32 simulated datasets. Our method consistently achieves higher median PCC values compared with Tangram, GraphST, and MACD across nearly all datasets, indicating an improved ability in accurately reconstructing spatial transcriptomic profiles. The notably narrower interquartile ranges and reduced variability associated with our method highlight its consistent and reliable performance across diverse biological contexts. This systematic improvement in correlation underscores the methodological advantage of S$^{2}$potAE, emphasizing its potential to reliably capture subtle yet meaningful biological differences across various tissue types and experimental conditions. Collectively, these results highlight S$^{2}$potAE’s capability in providing precise and biologically meaningful spatial reconstructions, essential for advancing ST research.

### Comprehensive analysis on model components and convergence behavior

To better understand the contributions of various components in S$^{2}$potAE, we conducted a systematic ablation study on three datasets: 10x_HBC, 10x_MBA, and DLPFC (slice 151673). As shown in Table 3, we examined the performance impact of removing or including specific modules, namely the SCE and the Spot-level Feature Extraction (SFE), as well as additional loss functions ($\mathcal{L}_{hist}$ and $\mathcal{L}_{match}$). The performance was evaluated using three key metrics: PCC, SSIM, and RMSE.

**Table 3:** Ablation study for inclusion of different components in 10x_HBC, 10x_MBA, and DLPFC (slice 151673) datasets. Improvements compared with the baseline are shown in italic.

Ablative models	10x_HBC			10x_MBA			DLPFC (slice 151673)		
	PCC$\uparrow $	SSIM$\uparrow $	RMSE$\downarrow $	PCC$\uparrow $	SSIM$\uparrow $	RMSE$\downarrow $	PCC$\uparrow $	SSIM$\uparrow $	RMSE$\downarrow $
Baseline	0.1774	0.0894	1.3654	0.1845	0.0665	1.3784	0.1654	0.0342	1.4328
Baseline w/ SCE	0.2154	0.2154	1.1844	0.2215	0.1721	1.2322	0.2176	0.1677	1.3654
*Improvement*	*+0.0380*	*+0.1260*	*-0.1810*	*+0.0370*	*+0.1056*	*-0.1462*	*+0.0522*	*+0.1335*	*-0.0674*
Baseline w/ SFE	0.1984	0.1440	1.2354	0.2010	0.1325	1.2897	0.2103	0.1543	1.3822
*Improvement*	*+0.0210*	*+0.0546*	*-0.1300*	*+0.0165*	*+0.0660*	*-0.0887*	*+0.0449*	*+0.1201*	*-0.0506*
Baseline w/ $\mathcal{L}_{hist}$	0.2015	0.1651	1.2102	0.2088	0.1458	1.2684	0.2023	0.1623	1.3778
*Improvement*	*+0.0241*	*+0.0757*	*-0.1552*	*+0.0243*	*+0.0793*	*-0.1100*	*+0.0369*	*+0.1281*	*-0.0550*
Baseline w/ $\mathcal{L}_{match}$	0.1865	0.1022	1.2357	0.1984	0.1441	1.2784	0.1923	0.1332	1.4182
*Improvement*	*+0.0091*	*+0.0128*	*-0.1297*	*+0.0139*	*+0.0776*	*-0.1000*	*+0.0269*	*+0.0990*	*-0.0146*

**Module ablation: the Role of SCE and SFE.** To further elucidate the contributions of each model component, we conducted ablation experiments assessing both the SCE and the SFE module in Table 3. The inclusion of the SCE led to significant improvements across all datasets, as evidenced by notable increases in PCC and SSIM, and reductions in RMSE, underscoring the importance of capturing spatial context for accurate spot deconvolution. Importantly, the addition of the SFE module, which extracts hierarchical spatial features from histological images, also resulted in consistent performance gains across all three tested datasets (10x_HBC, 10x_MBA, and DLPFC), all of which are derived from OCT-preserved tissues. For example, on the DLPFC dataset, incorporation of the SFE module increased PCC by 0.0449, improved SSIM by 0.1201, and decreased RMSE by 0.0506. Similar trends were observed for the other OCT datasets. These results demonstrate that, despite the relatively lower image quality of OCT compared with FFPE, the SFE module is able to extract meaningful spatial information that enhances model performance and facilitates the resolution of complex local heterogeneity.

**Loss function ablation: the impact of $\mathcal{L}_{hist}$ and $\mathcal{L}_{match}$.** By leveraging the shared encoder features, $\mathcal{L}_{hist}$ helps regularize the model and improves the representation of pathological characteristics. As shown in Table 3, incorporating the auxiliary pathological classification loss ($\mathcal{L}_{hist}$) consistently improves model performance across all evaluated datasets. Specifically, the inclusion of $\mathcal{L}_{hist}$ results in higher PCC and SSIM, along with lower RMSE, compared with the baseline model without this loss. For example, on the 10x_HBC dataset, PCC increases from 0.1774 to 0.2015 and SSIM rises from 0.0894 to 0.1651, while RMSE decreases from 1.3654 to 1.2102. Similar trends are observed in both the 10x_MBA and DLPFC datasets. These improvements indicate that the auxiliary pathological classification task serves as an effective regularization mechanism, guiding the model to learn more biologically meaningful and spatially coherent representations. The domain matching loss $\mathcal{L}_{match}$ was found to improve generalizability, particularly in cases where the datasets exhibited strong domain-specific variations. This demonstrates the importance of mitigating domain-specific biases to achieve biologically faithful deconvolution.

**Figure 4:**
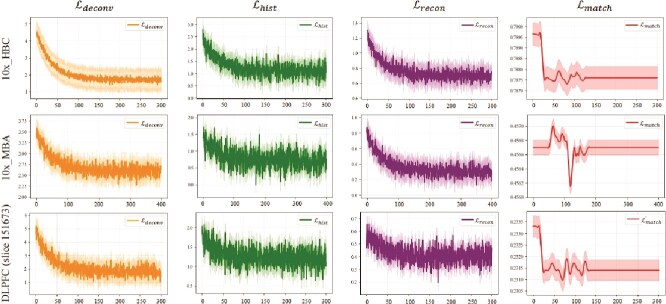
Convergence curves of four losses ($\mathcal{L}_{deconv}$, $\mathcal{L}_{hist}$, $\mathcal{L}_{recon}$, and $\mathcal{L}_{match}$) across three ST datasets. The solid lines represent mean loss values, and the shaded regions indicate standard deviations over multiple training runs. Stable and consistent declines in loss values demonstrate robust and effective joint optimization of the proposed S$^{2}$potAE model across diverse biological scenarios.

**Convergence guarantees for multiple module settings.** To assess the stability and robustness of S$^{2}$potAE, we visualized convergence curves for its four loss components across three datasets: 10x_HBC, 10x_MBA, and DLPFC (slice 151673) (Fig. 4). Biologically, the rapid and stable decrease of the deconvolution loss ($\mathcal{L}_{deconv}$) demonstrates that the model efficiently learns cell-type compositions from spatial signals. The auxiliary pathological classification loss ($\mathcal{L}_{hist}$) and reconstruction loss ($\mathcal{L}_{recon}$) both decline smoothly, indicating effective encoding of pathological features and faithful reconstruction of spatial expression patterns. The adversarial domain matching loss ($\mathcal{L}_{match}$) initially fluctuates but soon stabilizes, confirming that domain-invariant features are successfully extracted for better generalization across datasets.

Overall, these consistent convergence patterns across multiple ST datasets demonstrate the robustness of S$^{2}$potAE. The joint optimization of multiple biologically meaningful objectives ensures reliable and generalizable model performance for spatial transcriptomic analyses.

### Pathological region of interest

**Figure 5:**
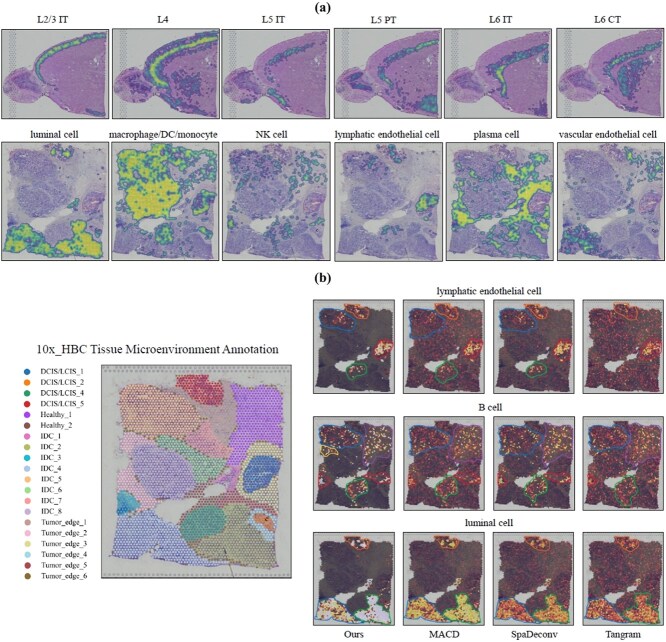
(a) **Pathological ROI:** visualization of attention-based spot-wise annotation for ST, highlighting the cellular distribution and microenvironmental features within tissue sections. Distinct cell populations (including luminal cells, macrophage/DC/monocytes, lymphatic and vascular endothelial cells, plasma cells, NK cells, and B cells) are shown in their respective spatial contexts, enabling detailed biological interpretation of the tissue. (b) **Comparison of cell-type spatial localization in BRCA tissue microenvironment.** Left: annotated BRCA tissue microenvironment showing distinct regions labeled as DCIS/LCIS, healthy, IDC, and tumor edge, with each region represented by a unique color. Right: spatial distribution predictions of lymphatic endothelial cells (top row), B cells (middle row), and luminal cells (bottom row) using four methods: Ours, MACD, SpaDeconv, and Tangram. The predicted cell-type localizations are outlined and overlaid on the tissue image for visual comparison with the annotated regions.

To further understand the spatially resolved cellular microenvironment and validate the efficacy of our proposed model, we dissected the pathological regions of interest (ROIs) as visualized in Fig. 5 (a). The annotation of these pathological ROIs was performed based on region boundaries provided in the original dataset; these boundaries were determined independently of our method and were not influenced by any model predictions. All ROI annotations were used exclusively for *post hoc* evaluation and visualization, not for model training or parameter tuning. Our analysis reveals that distinct immune and stromal cell populations are differentially localized across various tissue compartments, such as tumor core, boundary, and peritumoral stroma. Notably, luminal cells are enriched in epithelial regions, while macrophage/dendritic cell/monocyte clusters predominantly localize to stromal and immune-infiltrated areas. Endothelial cell subtypes (including lymphatic and vascular endothelial cells) highlight the spatial organization of the vasculature and lymphatic drainage, which are crucial for understanding tumor progression and metastasis. The presence and spatial distribution of plasma cells, NK cells, and B cells provide additional layers of insight into local immune surveillance and response. After model training, we applied our S$^{2}$potAE framework to perform spot-wise deconvolution, generating predicted cell-type proportions for each spot based solely on the input expression profiles and the deconvolution loss ($\mathcal{L}_{{deconv}}$). For visualization, these predictions were then mapped back onto the tissue image using each spot’s spatial coordinates, and the predefined pathological region boundaries were overlaid to facilitate direct comparison. This approach allows us to intuitively assess the spatial correspondence between predicted cellular composition and the underlying pathological architecture.

Crucially, our model leverages the proposed $\mathcal{L}_{{recon}}$ loss to robustly reconstruct these spatially heterogeneous cell populations, thereby providing a stronger supervision signal for $\mathcal{L}_{{deconv}}$. This joint optimization ensures that the deconvolution results are both accurate and biologically meaningful, as $\mathcal{L}_{{recon}}$ encourages faithful representation of local transcriptomic profiles and $\mathcal{L}_{{deconv}}$ ensures correct cell-type proportion estimation. Further, the integration of the $\mathcal{L}_{{hist}}$ loss incorporates high-level image-derived features, facilitating a richer understanding of the visual context and promoting the model’s capacity to align molecular and morphological information.

### Spatial mapping of immune and epithelial cell types in 10x_HBC tissue

To further elucidate the spatial organization of key cell populations within the 10x_HBC microenvironment, we visualized the annotated tissue microenvironment as shown in Fig. 5 (b). The tissue section was segmented into distinct regions, including DCIS/LCIS, IDC, healthy tissue, and tumor edges, providing a comprehensive map of the tumor and its surrounding microenvironment. Our spatial analysis demonstrates that lymphatic endothelial cells are predominantly localized at the periphery of tumor regions and, within specialized vascular niches, aligning with their roles in lymphatic drainage and immune cell trafficking. B cells are observed to be enriched at the tumor boundary and in adjacent stromal compartments, suggesting active immune surveillance and potential formation of tertiary lymphoid structures. Luminal epithelial cells are densely concentrated within the epithelial cores of tumor and premalignant regions, consistent with the known histological architecture of BRCA. This compartmentalization underscores the spatial heterogeneity of cell populations and highlights the intricate interplay between malignant, stromal, and immune elements within the tissue. Notably, our method uniquely identifies the boundary localization of B cells at the region corresponding to “tumor_edge_2” in the ground truth annotation. Unlike competing approaches, only our model accurately delineates this B cell-enriched boundary, highlighting its advanced capability to resolve fine-grained microenvironmental structures that are critical for understanding immune–tumor interactions. This precise mapping of B cell boundaries is absent in the results of MACD, SpaDeconv, and Tangram, which fail to capture the distinct compartmentalization present in the annotated ground truth. By mapping these cell types with high resolution, our analysis provides crucial insights into the tissue microenvironment, informing hypotheses about local immune response, tumor progression, and therapeutic resistance.

### Cross-platform and further biological validation

To comprehensively evaluate the robustness and generalizability of our method across different ST platforms and biological contexts, we conducted additional experiments on Slide-seqV2 Hippocampus and MERFISH Hypothalamus datasets. These datasets represent distinct technical frameworks and tissue architectures, enabling stringent validation of S$^{2}$potAE’s performance.

**Table 4:** Quantitative performance of the proposed model under *in silico* artifact perturbation on 10x_HBC and 10x_MBA datasets.

Artifact type	10x_HBC			10x_MBA		
	PCC$\uparrow $	SSIM$\uparrow $	RMSE$\downarrow $	PCC$\uparrow $	SSIM$\uparrow $	RMSE$\downarrow $
Original image	0.2441	0.2541	1.1032	0.2498	0.2015	1.1215
Crack	0.2203	0.2284	1.2822	0.2140	0.1774	1.2742
Fold	0.2405	0.2483	1.1492	0.2441	0.1950	1.1482
Stain	0.2118	0.2259	1.2528	0.2139	0.1751	1.2349
Crack_Fold_Stain	0.2089	0.2117	1.3271	0.1964	0.1672	1.3177

**Figure 6:**
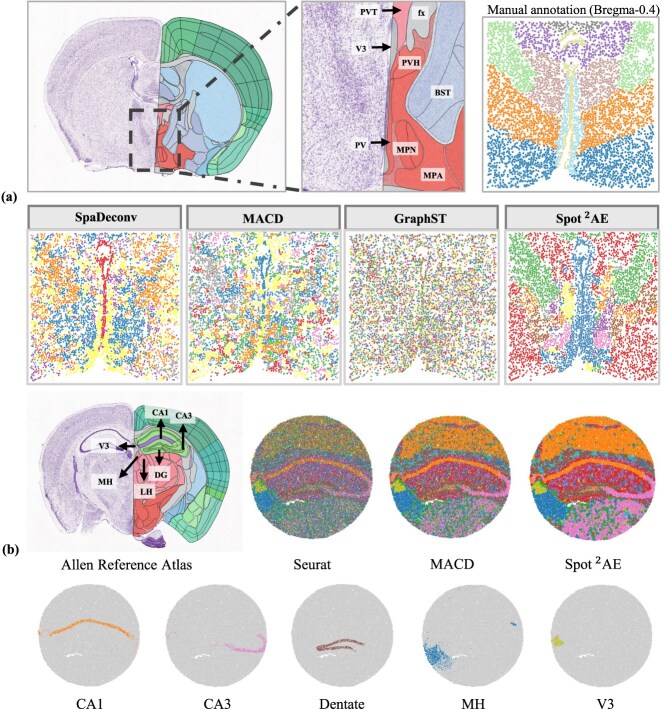
(a) Anatomical context is established by the Allen Reference Atlas (top left), which delineates the organization of the hypothalamus. A magnified view (top middle) highlights the fine structure and precise boundaries of individual subregions. Manual annotation based on expert curation (top right) serves as the reference standard for evaluating automated methods. (b) The Allen Reference Atlas (left) provides anatomical delineation of key hippocampal subregions, including CA1, CA3, Dentate Gyrus (DG), medial habenula (MH), and V3. The middle panels display spatial domain identification results from Seurat, MACD, and S$^{2}$potAE using the same input data and parameters. The bottom row highlights key spatial domains identified by S$^{2}$potAE.

We first assessed our method on a MERFISH dataset derived from the mouse hypothalamic preoptic area at Bregma –0.04 mm, with the aim of evaluating its ability to resolve fine-scale anatomical structures in high-resolution data. As shown in Fig. 6 (a), the anatomical context is provided by the Allen Reference Atlas (top left), which delineates the organization of the hypothalamus. A magnified view (top middle) highlights the fine structure and precise boundaries of individual regions, while manual annotation based on expert curation (top right) serves as the reference standard for evaluation. By comparing the deconvolution results of four methods, we assessed their capacity to distinguish closely adjacent subregions. Notably, S$^{2}$potAE demonstrates clear superiority in accurately capturing the spatial boundaries of regions such as the MPN and MPA, where other methods, including SpaDeconv, MACD, and GraphST, fail to resolve these fine structures. The clusters generated by S$^{2}$potAE closely match manual annotations, exhibiting minimal mixing between adjacent regions and preserving the compactness of even the smallest microenvironments. These results highlight S$^{2}$potAE’s strength in resolving intricate anatomical domains and demonstrate its advantage over existing approaches in high-resolution scenarios.

To further test the applicability of S$^{2}$potAE to single-cell ST technologies, we next evaluated its performance on a mouse hippocampus dataset generated with Slide-seqV2 (Puck_200115_08). This experiment was designed to assess the method’s ability to delineate cell types and anatomical subregions in data with near single-cell resolution and considerable expression sparsity. As summarized in Fig. 6 (B), Seurat produces diffuse and intermixed clusters that lack clear anatomical correspondence, and MACD yields only partially improved boundaries. In contrast, S$^{2}$potAE achieves precise and cohesive delineation of hippocampal subregions, with boundaries that closely match the anatomical reference. Our method is particularly effective in distinguishing challenging regions such as CA3 and MH, generating spatial domains that are less fragmented and more biologically coherent. This capability is essential for studying region-specific features in complex tissues. Collectively, these experiments provide strong evidence that S$^{2}$potAE generalizes well across a variety of ST technologies and tissue architectures, supporting its utility in both research and clinical applications.

### Robustness evaluation under *in silico* artifact perturbations

**Figure 7:**
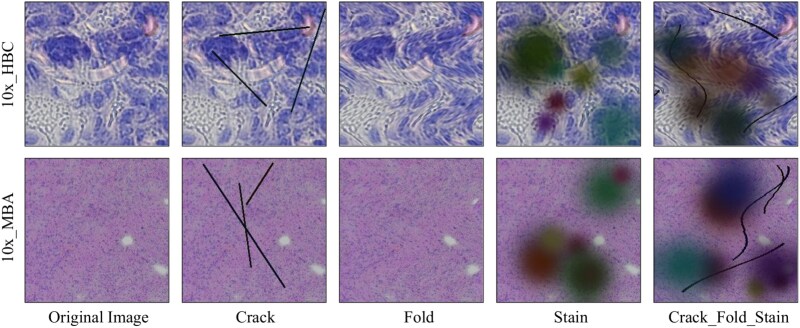
Representative examples of *in silico* artifact perturbations applied to H&E images from two datasets (10x_HBC and 10x_MBA). From left to right: original image, crack, fold, stain, and combined crack_fold_stain perturbations.

To comprehensively evaluate the robustness of our model, we performed comprehensive *in silico* perturbation experiments to systematically simulate common artifacts observed in hematoxylin and eosin (H&E) stained histological images. These include cracks, tissue folds, staining irregularities, and their combinations. As illustrated in Fig. 7, the simulated artifacts were applied to both datasets used in this study (10x_HBC and 10x_MBA). The perturbation procedures were implemented algorithmically to mimic realistic experimental imperfections in image acquisition and tissue preparation, thereby enabling a controlled evaluation of model robustness.

For a rigorous quantitative assessment, we evaluated our model’s performance under each perturbation condition using PCC, SSIM, and RMSE. The results are summarized in Table 4. Three common artifact types were examined individually (crack, fold, stain) and in combination (crack_fold_stain). The model demonstrated stable and reliable performance under individual perturbations. For instance, on the 10x_HBC dataset, the PCC decreased only marginally from 0.2441 (original) to 0.2203 (crack), 0.2405 (fold), and 0.2118 (stain), with SSIM and RMSE showing similarly small variations. A consistent trend was observed on the 10x_MBA dataset. A more notable performance decline occurred only under the combined perturbation condition, where PCC decreased to 0.2089 (HBC) and 0.1964 (MBA), accompanied by a moderate RMSE increase. Despite these compounded effects, the model retained strong predictive capacity, indicating robust performance even in the presence of severe imaging imperfections. Overall, these findings provide quantitative and visual evidence that our model exhibits strong resilience to common experimental artifacts in histological imaging. This robustness highlights its practical utility and generalizability for ST applications, where such imperfections are frequently encountered in real-world scenarios.

### Discussion: architectural basis for robustness in S$^{2}$potAE

The robustness of S$^{2}$potAE arises from its explicit multimodal regularization framework, rather than from any single architectural component. By jointly leveraging spatial transcriptomic, spatial coordinate, and histological image features, the model integrates complementary sources of biological information. This multimodal fusion not only enriches representation learning but also acts as a natural safeguard against overfitting to modality-specific noise or artifacts, as the model is never solely reliant on one data type. Further, the use of auxiliary tasks and domain adaptation serves as additional regularization, encouraging the model to learn biologically meaningful and domain-invariant features. These design choices (motivated by the need for generalizability) ensure that S$^{2}$potAE maintains stable performance across varied datasets, tissue types, and technical conditions. In summary, the architecture of S$^{2}$potAE is purposefully constructed to promote robustness through multimodal integration and regularization, proactively addressing concerns of overfitting and domain shift. We appreciate the reviewer’s recommendation, and believe this discussion provides a clearer scientific rationale for the observed generalizability of our model.

## Conclusion

In this study, we have proposed S$^{2}$potAE, an innovative spatial autoencoder framework specifically designed for the accurate deconvolution of spot-level ST data. To the best of our knowledge, this is the first unified deep-learning framework to systematically integrate spatial coordinates, histological image features, and transcriptomic profiles to enhance cellular composition inference. By incorporating a multilevel aggregation strategy, our method effectively captures complex spatial dependencies and morphological characteristics inherent in heterogeneous tissues.

S$^{2}$potAE prominently features an SCE leveraging graph neural networks to model spatial relationships among transcriptomic spots, significantly improving the biological coherence of inferred spatial patterns. Additionally, we introduce a perceptual feature extraction module that extracts informative visual embeddings from histology images, providing critical morphological context to guide the reconstruction of gene expression profiles. Furthermore, the integration of an auxiliary pathological classification task serves as an effective regularization mechanism, improving model interpretability and biological relevance.

Through extensive evaluations on diverse simulated datasets and challenging real-world datasets—including human breast cancer, mouse brain anterior, and human dorsolateral prefrontal cortex—we demonstrate that S$^{2}$potAE consistently outperforms contemporary methods across multiple quantitative and qualitative metrics. These results underscore our model’s capacity to accurately dissect cellular heterogeneity, precisely delineate tumor boundaries, and robustly handle complex tissue architectures.

Looking forward, S$^{2}$potAE can serve as a foundational methodology for ST analysis, facilitating deeper biological insights into tissue organization and pathology. Further research efforts could explore extending our framework to multi-omics integration, domain adaptation across diverse biological systems, and scaling to higher resolution spatial data, providing even broader applicability in biological and clinical research scenarios.

**Key Points**

- We propose S$^{2}$potAE, a novel deep-learning-based spatial spot autoencoder that integrates transcriptomic data, spatial coordinates, and histological image features, significantly improving the accuracy and interpretability of spatial transcriptomics deconvolution.
- Our framework employs a graph neural network-based spatial context encoder, effectively modeling spatial relationships between transcriptomic spots and preserving biological coherence.
- The spot-level feature extraction and fusion module utilizes multi-scale histological image patches, providing critical morphological context that substantially enhances cellular composition inference.
- An auxiliary pathological classification task is introduced to regularize the model, enhancing representation learning and biological interpretability.
- Extensive benchmarking on simulated and real-world datasets demonstrates superior performance of S$^{2}$potAE over existing methods, highlighting accurate cellular composition prediction, precise tumor boundary identification, and robust generalization capabilities.

## Data Availability

The implementation of S$^{2}$potAE is available at https://github.com/bravotty/S2potAE.

## References

[1] Moffitt JR, Zhuang X. RNA imaging with multiplexed error-robust fluorescence in situ hybridization (MERFISH). Methods Enzymol 2016;572:1–49. 10.1016/bs.mie.2016.03.020. 27241748 PMC5023431

[2] Shah S, Takei Y, Zhou W. et al. Dynamics and spatial genomics of the nascent transcriptome by intron seqFISH. *Cell* 2018;174:363–376.e16. 10.1016/j.cell.2018.05.035 29887381 PMC6046268

[3] Lugmayr W, Kotov V, Goessweiner-Mohr N. et al. StarMap: a user-friendly workflow for Rosetta-driven molecular structure refinement. *Nat Protoc* 2023;18:239–64. 10.1038/s41596-022-00757-9 36323866

[4] Thind AS, Monga I, Thakur PK. et al. Demystifying emerging bulk RNA-seq applications: the application and utility of bioinformatic methodology. *Brief Bioinform* 2021;22:bbab259. 34329375 10.1093/bib/bbab259

[5] Muzellec B, Teleńczuk M, Cabeli V. et al. PyDESeq2: a python package for bulk RNA-seq differential expression analysis. *Bioinformatics* 2023;39:btad547. 37669147 10.1093/bioinformatics/btad547 PMC10502239

[6] Cobos FA, Panah MJN, Epps J. et al. Effective methods for bulk RNA-seq deconvolution using scnRNA-seq transcriptomes. *Genome Biol* 2023;24:177. 10.1186/s13059-023-03016-6 PMC10394903 37528411

[7] Dar D, Dar N, Cai L. et al. Spatial transcriptomics of planktonic and sessile bacterial populations at single-cell resolution. *Science* 2021;373:eabi4882. 10.1126/science.abi4882 PMC8454218 34385369

[8] Chen A, Liao S, Cheng M. et al. Spatiotemporal transcriptomic atlas of mouse organogenesis using DNA nanoball-patterned arrays. *Cell* 2022;185:1777–1792.e21. 10.1016/j.cell.2022.04.003 35512705

[9] Larsson L, Frisén J, Lundeberg J. Spatially resolved transcriptomics adds a new dimension to genomics. *Nat Methods* 2021;18:15–8. 10.1038/s41592-020-01038-7 33408402

[10] Stickels RR, Murray E, Kumar P. et al. Highly sensitive spatial transcriptomics at near-cellular resolution with Slide-seqV2. *Nat Biotechnol* 2021;39:313–9. 10.1038/s41587-020-0739-1 33288904 PMC8606189

[11] Moffitt JR, Bambah-Mukku D, Eichhorn SW. et al. Molecular, spatial, and functional single-cell profiling of the hypothalamic preoptic region. *Science* 2018;362:eaau5324. 10.1126/science.aau5324 PMC6482113 30385464

[12] Pham D, Tan X, Balderson B. et al. Robust mapping of spatiotemporal trajectories and cell–cell interactions in healthy and diseased tissues. *Nat Commun* 2023;14:7739. 38007580 10.1038/s41467-023-43120-6 PMC10676408

[13] Fischer DS, Schaar AC, Theis FJ. Modeling intercellular communication in tissues using spatial graphs of cells. *Nat Biotechnol* 2023;41:332–6. 10.1038/s41587-022-01467-z 36302986 PMC10017508

[14] Rodriques SG, Stickels RR, Goeva A. et al. Slide-seq: a scalable technology for measuring genome-wide expression at high spatial resolution. *Science* 2019;363:1463–7. 30923225 10.1126/science.aaw1219 PMC6927209

[15] Wang X, He Y, Zhang Q. et al. Direct comparative analyses of 10x genomics chromium and smart-seq2. *Genomics Proteomics Bioinf* 2021;19:253–66. 10.1016/j.gpb.2020.02.005 PMC8602399 33662621

[16] Kleshchevnikov V, Shmatko A, Dann E. et al. Cell2location maps fine-grained cell types in spatial transcriptomics. *Nat Biotechnol* 2022;40:661–71. 10.1038/s41587-021-01139-4 35027729

[17] Biancalani T, Scalia G, Buffoni L. et al. Deep learning and alignment of spatially resolved single-cell transcriptomes with tangram. *Nat Methods* 2021;18:1352–62. 10.1038/s41592-021-01264-7 34711971 PMC8566243

[18] Li X, Zhu F, Min W. SpaDiT: diffusion transformer for spatial gene expression prediction using scRNA-seq. *Brief Bioinform* 2024;25:bbae571. 39508444 10.1093/bib/bbae571 PMC11541600

[19] Min W, Shi Z, Zhang J. et al. Multimodal contrastive learning for spatial gene expression prediction using histology images. *Brief Bioinform* 2024;25:bbae551. 39471412 10.1093/bib/bbae551 PMC11952928

[20] Lopez R, Li B, Keren-Shaul H. et al. DestVI identifies continuums of cell types in spatial transcriptomics data. *Nat Biotechnol* 2022;40:1360–9. 10.1038/s41587-022-01272-8 35449415 PMC9756396

[21] Hao X, Wang S, Fang M. et al. SPACEL: deep learning-based characterization of spatial transcriptome architectures. *Nat Commun* 2023;14:7603. 37990022 10.1038/s41467-023-43220-3 PMC10663563

[22] Coleman K, Jian H, Schroeder A. et al. SpaDecon: cell-type deconvolution in spatial transcriptomics with semi-supervised learning. *Commun Biol* 2023;6:378. 37029267 10.1038/s42003-023-04761-x PMC10082183

[23] Wang F, Yang F, Huang L. et al. Deep domain adversarial neural network for the deconvolution of cell type mixtures in tissue proteome profiling. *Nat Mach Intell* 2023;5:1236–49. 10.1038/s42256-023-00737-y

[24] Lin H, Liu X, Wang S. et al. Masked adversarial neural network for cell type deconvolution in spatial transcriptomics. IEEE International Conference on Bioinformatics and Biomedicine (BIBM). IEEE, 2024;2024:619–22.

[25] Swain AK, Pandit V, Sharma J. et al. SpatialPrompt: spatially aware scalable and accurate tool for spot deconvolution and domain identification in spatial transcriptomics. *Commun Biol* 2024;7:639. 38796505 10.1038/s42003-024-06349-5 PMC11127982

[26] Long Y, Ang KS, Li M. et al. Spatially informed clustering, integration, and deconvolution of spatial transcriptomics with GraphST. *Nat Commun* 2023;14:1155. 10.1038/s41467-023-36796-3 36859400 PMC9977836

[27] Chen RJ, Ding T, Lu MY. et al. Towards a general-purpose foundation model for computational pathology. *Nat Med* 2024;30:850–62. 10.1038/s41591-024-02857-3 PMC11403354 38504018

[28] Vaswani A, Shazeer N, Parmar N. et al. Attention is all you need. In: Guyon I, Luxburg UV, Bengio S, Wallach H, Fergus R, Vishwanathan S, Garnett R (eds) *Advances in Neural Information Processing Systems* 30 (NeurIPS 2017). Curran Associates, Inc., Red Hook, NY, USA, 2017.

[29] Chen T, Wei X, Xie L. et al. SELF-Former: multi-scale gene filtration transformer for single-cell spatial reconstruction. *Brief Bioinform* 2024;25:bbae523. 39413798 10.1093/bib/bbae523 PMC11483138

[30] Li B, Zhang W, Guo C. et al. Benchmarking spatial and single-cell transcriptomics integration methods for transcript distribution prediction and cell type deconvolution. *Nat Methods* 2022;19:662–70. 10.1038/s41592-022-01480-9 35577954

[31] Li M, Zhang X, Ang KS. et al. DISCO: a database of deeply integrated human single-cell omics data. *Nucleic Acids Res* 2022;50:D596–602. 10.1093/nar/gkab1020 34791375 PMC8728243

[32] Maynard KR, Collado-Torres L, Weber LM. et al. Transcriptome-scale spatial gene expression in the human dorsolateral prefrontal cortex. *Nat Neurosci* 2021;24:425–36. 10.1038/s41593-020-00787-0 33558695 PMC8095368

[33] Li Z, Zhou X. BASS: multi-scale and multi-sample analysis enables accurate cell type clustering and spatial domain detection in spatial transcriptomic studies. *Genome Biol* 2022;23:168. 10.1186/s13059-022-02734-7 35927760 PMC9351148

